# Unravelling the diversity of pacing behaviours in adults with chronic conditions: a cross-sectional study

**DOI:** 10.1136/bmjopen-2025-104566

**Published:** 2026-05-27

**Authors:** Ioulia Barakou, Bregje L Seves, Ulric Abonie, Tracy Finch, Katie Hackett, Florentina Hettinga

**Affiliations:** 1 Department of Nursing, Midwifery & Health, Northumbria University, Newcastle upon Tyne, UK; 2 Department of Human Movement Sciences, University Medical Center Groningen, Groningen, Netherlands; 3 Department of Sport Exercise and Rehabilitation, Northumbria University, Newcastle upon Tyne, UK; 4 Department of Social Work, Education and Community Wellbeing, Northumbria University, Newcastle upon Tyne, UK; 5 Department of Human Movement Sciences, Vrije Universiteit Amsterdam, Amsterdam, Netherlands

**Keywords:** Fatigue, Chronic Disease, Exercise, Behavior

## Abstract

**Abstract:**

**Objectives:**

This study aimed to (1) examine if activity fluctuations over 1 week differ between two groups: individuals who have received fatigue management advice versus those who have not, (2) examine the associations between activity fluctuations and fatigue, engagement in pacing, perceived risk of overactivity, quality of life, self-regulation and physical activity (PA) and if they differ for the two groups and (3) explore whether there are distinct pacing patterns across the week in adults with chronic conditions.

**Design:**

Exploratory cross-sectional observational study.

**Setting:**

Participants were recruited from a fatigue management clinic in the UK and through university networks.

**Participants:**

29 adults with chronic conditions who experience fatigue (18 received fatigue management advice; 11 did not).

**Primary and secondary outcome measures:**

The primary outcome was activity fluctuations (SD of accelerometer-derived vector magnitude counts per minute), measured using the ActiGraph wGT3X-BT over 7 consecutive days. Secondary outcomes included fatigue (Fatigue Severity Scale), engagement in activity pacing, perceived risk of overactivity, quality of life (Functional Assessment of Cancer Therapy-General Instrument), self-regulation of PA (Physical Activity Self-Regulation scale) and self-reported PA (International Physical Activity Questionnaire-short form).

**Results:**

No difference in activity fluctuations during the day was found between groups. Associations between self-regulation of PA and device-based PA with activity fluctuations significantly differed between groups (respectively, standardised regression (β)=1686.14; p=0.006 and β=288.83; p=0.042). Five distinct activity pacing patterns were identified through visual inspection of individual activity profiles: (1) high fluctuations in the morning, (2) high fluctuations in the afternoon, (3) high fluctuations at two time points, (4) consistent pacing pattern and (5) varied pacing patterns.

**Conclusions:**

Individuals who received fatigue management advice demonstrated significant associations between activity fluctuations and self-regulation, suggesting a goal-directed approach, which could indicate that tailored support and goal setting could help in balancing rest and activity. This study also identified five distinct activity pacing patterns in individuals with chronic conditions, emphasising the need for tailored fatigue management instead of a ‘one-size-fits-all’ approach.

**Trial registration number:**

NCT06001970.

**STRENGTHS AND LIMITATIONS OF THIS STUDY**

- Accelerometry was used to objectively measure activity fluctuations across different time periods of the day over seven consecutive days.
- Validated instruments were used to assess fatigue, quality of life, self-regulation of physical activity and engagement in pacing.
- The small sample size (n=29) limits the generalisability of the findings and the statistical power to detect between-group differences.
- Participants recruited through fatigue clinics and university networks who had received fatigue management advice may have received this from different healthcare providers.

## Background

 Individuals with a wide range of chronic conditions often experience fatigue,^1^ necessitating the pacing of their activities to manage their fatigue and sustain daily life physical activity (PA) engagement.^2 3^ To manage their fatigue while maintaining engagement in daily life activities, adults with chronic conditions have reported the need for enhanced support and guidance in balancing rest and PA appropriately, which can be facilitated through a process called activity pacing.^3^,^5^ Activity pacing is a behavioural approach to achieve sustainable self-regulated exercise and activity engagement while coping with fatigue, optimise PA levels, and maximise health and well-being.^3 6 7^ Sustained PA is crucial for managing chronic conditions, improving overall health and alleviating fatigue.^8 9^ Rehabilitation studies have demonstrated that targeted exercise interventions could improve symptomology in chronic conditions, including improvements in breathlessness, quality of life and functional capacity following inspiratory muscle training in post-COVID-19 recovery^10^ and exercise-based rehabilitation programmes.^11^ Although activity pacing can be an important mechanism for maintaining PA engagement, reducing fatigue, and enhancing overall health-related quality of life (HRQoL),^4 6 12^ it is unclear how people with chronic conditions pace their daily life activities throughout the day and week. This gap in understanding underscores the need for further research, to provide insights underpinning further development of activity pacing support and guidance that healthcare professionals could include in their treatment to help individuals manage their fatigue, health and well-being.^3 6^

Activity pacing interventions typically involve breaking down daily tasks to manage fatigue while increasing or maintaining consistent activity levels.^36 13^,^15^ A recent conceptual model emphasised the multidimensionality of pacing, including social environment, anxiety/depression, restful activities, and self-regulation, beyond just fatigue and PA.^6^ Due to this multidimensional nature and the need to tailor pacing to each individual’s specific needs and goals, there are currently no standardised concrete steps for its administration. Moreover, pacing can be assessed in different ways, ranging from device-based measures using accelerometry to subjective self-management approaches such as Spoon Theory, where individuals budget their perceived energy across daily tasks.^16^ Furthermore, previous studies on pacing in osteoarthritis and multiple sclerosis have demonstrated benefits such as reduced fatigue and improved PA levels without worsening fatigue.^2 17^ However, the evidence for activity pacing is not consistent across all conditions. A recent meta-analysis of pacing in ME/CFS found that while pacing exerted a large beneficial effect on fatigue, effects on physical function and pain were trivial.^18^ While these studies provide important insights into the benefits of pacing, there is a need to better understand typical interindividual and intraindividual variations in pacing patterns, to be able to tailor and individualise advice and recommendations regarding pacing and fatigue management.^14^ One aspect that remains underexplored is the occurrence of activity fluctuations in PA patterns throughout the day (morning, afternoon and evening). Activity fluctuations refer to the variability in PA intensity, reflecting how an individual distributes their activity across different time periods. These activity fluctuations can be described and better understood by measuring activity counts.^12 19^ More insights into activity fluctuations could offer a novel perspective on the ways individuals regulate their activity throughout the day. It could be crucial for developing tailored pacing interventions that help maintain consistent PA while managing fatigue.

To address the complexity of fatigue across various chronic conditions, this study adopts a transdiagnostic approach, recognising that fatigue is common across different conditions, including fibromyalgia and multiple sclerosis.^1 6^ It also allows for the identification of common strategies that can be applied to manage fatigue, regardless of specific diagnoses as in some cases underlying causes remain undetermined.^8^ The increasing prevalence of multimorbidity, the presence of two or more chronic conditions,^20^ makes transdiagnostic approaches increasingly relevant for effectively addressing common symptoms such as fatigue. In this context, PA has been widely recognised as a beneficial approach for managing fatigue symptoms across chronic conditions.^8^ However, while the benefits of PA are well-documented, less is known about the typical activity pattern distribution throughout the day and week in persons with chronic conditions. Exploring activity patterns among different persons could provide insights into understanding fatigue management and PA engagement. This differentiation may reveal common pacing patterns that could inform the development of tailored interventions aimed at supporting PA engagement and addressing fatigue across various conditions. These insights would also enable healthcare professionals to develop more personalised approaches by gaining a better understanding of the range of activity profiles individuals with chronic conditions engage in, and how these profiles relate to quality of life and other health outcomes. Although exercise regulation has been studied in sports and exercise^21 22^ ; there is a gap in understanding how adults with chronic conditions pace their daily and weekly activities, managing their fatigue symptoms and physical limitations. Exploring pacing over longer timeframes in individuals with chronic conditions may offer new insights into exercise regulation and daily life functioning.

Further supporting the promise of pacing interventions, studies additionally show that individuals who had not received any pacing guidance or advice often experience stable but severe fatigue complaints and decreased PA levels.^13 23 24^ These findings suggest that individuals may struggle to manage their fatigue and PA without guidance,^4 5^ highlighting the importance of developing insights on how to optimise pacing advice. Self-regulation, defined as the ability to understand and manage behaviour and reactions to feelings, also includes planning and goal-setting abilities.^25^ It is an important factor in health behaviour theories and plays a significant role in exercise regulation and pacing.^6 26 27^ This is further supported by qualitative studies, where individuals with chronic conditions emphasised the critical role of activity pacing advice in managing fatigue and enabling them to engage in more activities throughout the day.^4 5^ Also, strategic and anticipatory pacing guidance has been shown to be effective in fatigue management.^12^ Adults with chronic conditions often lack clear fatigue management strategies when not provided with guidance from healthcare professionals.^28^ It was recently proposed that fatigue management should follow a multidimensional approach to address the various factors that contribute to fatigue and pacing.^24^ Another recent large-scale randomised controlled trial examining the efficacy of a digital platform with activity tracking and just-in-time energy management support in adults with long COVID found that, while the intervention was safe and feasible, it did not reduce the frequency or severity of post-exertional malaise compared with usual care.^29^ Alongside this, consumer-facing energy management applications have also emerged, though their suitability for research purposes remains limited. While some studies have explored activity pacing interventions,^12 17 30 31^ there is a need for further research to assess and understand the current effects of fatigue management advice.

To prepare for the development of a multidimensional pacing intervention, we will first conduct a cross-sectional study comparing individuals who had received fatigue management advice (in a fatigue clinic in the UK) versus those who have not. We will also explore the diversity of activity fluctuations among chronic conditions, which could provide novel insights into the process of activity pacing. Thus, this study aims to (1) examine whether activity fluctuations over a period of 1 week differ between individuals who had received fatigue management advice and those who have not; (2) examine whether the activity fluctuations are associated with fatigue, engagement in pacing, perceived risk of overactivity, HRQoL, self-regulation of PA and PA (self-reported and device-based) in all the participants and whether these associations differ for the two groups and (3) identify whether there are distinct pacing patterns across different days of the week for the participants. Our hypothesis was based on the expectation that individuals who had received fatigue management advice will engage in higher levels of PA.^12 17^ We also expect differences in activity fluctuations between the two groups. Furthermore, we anticipate that these fluctuations will provide insights into the balance between activity and rest. We also expect that the associations between the activity fluctuations and fatigue, perceived engagement in pacing, perceived risk of overactivity, self-regulation of PA, PA (self-reported and device-based) and HRQoL will be stronger in the group that received fatigue management advice, indicating a more goal-directed approach, alternating activities with periods of rest. Lastly, we hypothesised that there will be distinct pacing patterns in adults with chronic conditions,^6 24^ which can be clustered to inform the future tailoring of fatigue management approaches.

## Methods

### Design

In this cross-sectional study, all participants were informed about the study rationale, procedures, risks and benefits. The study has been registered at ClinicalTrials.gov (NCT06001970; Registration Date: 26 June 2023).

### Participants and procedures

This study used purposive sampling to include adults with chronic conditions who experience fatigue, divided into two groups based on whether they had received fatigue management advice prior to or during this study: (1) individuals who had received fatigue management advice (the advice group) and (2) individuals who had not received fatigue management advice (non-advice group). The advice group comprised individuals who received tailored fatigue management advice including activity pacing from a multidisciplinary team at a fatigue clinic, including health psychologists, physiotherapists, occupational therapists and a medical consultant. Participants were recruited through NHS fatigue clinics in Newcastle Upon Tyne in 2023, specialising in fatigue management including individuals who had received fatigue management advice (assigned to advice group) and those on the waiting list who have not received advice (assigned to non-advice group). Specifically, clinicians distributed an information letter to all eligible potential participants attending the fatigue clinics or on the waiting list; no additional selection criteria were applied beyond the study’s inclusion and exclusion criteria. An estimated 80–120 information sheet letters were distributed to potential participants through the fatigue clinics. Additionally, participants were recruited from the community in Newcastle Upon Tyne in 2023 through the university including both students and staff members, and they were asked if they had received fatigue management advice or not, to assign them to the corresponding group. The community recruitment process involved sending an email with information about the research study to all students and staff members at the university at the time. Participants recruited through University networks who reported having received fatigue management advice may have received this advice from different clinics or healthcare providers, and the content of the advice received may have not standardised across participants.

Potential participants in both cases were checked for the inclusion and exclusion criteria. They were included if they (1) were adults ≥18 years of age, (2) were diagnosed with any chronic condition, lasting 3 months or longer,^32^ (3) were ambulatory and (4) experienced chronic fatigue. Participants were excluded if they were not able to complete the study questionnaires online.

As this is an exploratory study and the first, to our knowledge, to examine device-based activity pacing behaviours in adults with chronic conditions, no formal a priori power calculation was performed.

Eligible and willing participants were evaluated by filling out a set of validated questionnaires digitally (questionnaires are provided in online supplemental file 1). First, participants were asked to answer demographic (eg, biological sex, age, employment status) and anthropometric (body mass and height) questions. Then, they filled out questionnaires on HRQoL, fatigue, engagement in pacing, perceived risk of overactivity, self-regulation of PA and self-reported PA. Additionally, participants were invited to wear an Actigraph for 7 full days.

### Outcome measures

#### Health-related quality of life

HRQoL of life was measured by the 27-item Functional Assessment of Cancer Therapy-General Instrument (FACT-G).^33^ FACT-G is validated in many clinical populations.^34 35^ The score ranges from 0 to 108 and higher scores indicate better HRQoL.

#### Fatigue

The severity of fatigue was measured by using the Fatigue Severity Scale (FSS), a 9-item questionnaire,^36^ which ranges from 1 to 7 (1=completely disagree; 7=completely agree). It is a validated and reliable scale to assess the impact of fatigue in various clinical populations.^37 38^ A higher score indicates greater fatigue severity, while a mean score of 4 or greater indicates severe fatigue.^39 40^

#### Activity Pacing and Risk of Overactivity Questionnaire

Activity pacing was measured by the Activity Pacing and Perceived Risk of Overactivity Questionnaire, which is a seven-item questionnaire developed by the ReSpAct team.^41^ Two different attitudes towards activity pacing are measured: engagement in activity pacing (five items; score range 5–25; higher score indicating highengagement in pacing) and perceived risk of overactivity (two items; score range 2–10; higher score indicates high risk of perceived overactivity) on a scale ranging from 1 to 5 (1 = never; 5 = very often). Seven items were scored and the sum scores were calculated for the two different attitudes. Reliability and construct validity have been investigated and first data on this have been previously published.^28 41^

#### Self-regulation of PA

Self-regulation of PA was measured by the Physical Activity Self-Regulation scale (PASR-12).^42^ PASR-12 includes six domains (two items each): self-monitoring, goal setting, eliciting social support, reinforcements, time management and relapse prevention. PASR-12 has demonstrated validity and reliability in adults.^43^ The score ranges from 1 to 5 (1=never; 5=very often) and total scores were calculated by summing up the scores of the items. Higher scores indicate greater use of self-regulatory strategies.

#### Self-reported PA

Self-reported PA was measured by the International Physical Activity Questionnaire-short form, which includes seven items.^44^ This questionnaire records the time spent on each PA within the last 7 days. These values were entered into a scoring protocol, to produce a continuous variable (eg, metabolic equivalent (MET) minutes per week), in which MET scores were calculated using the following values: low=3.3 METs, moderate PA=4.0 METs and vigorous PA=8.0 METs. It has been validated in different populations.^45 46^

#### Device-based PA

PA was assessed by the Actigraph WGT3X-BT (Pensacola, Florida, USA), which is a reliable, valid and widely accepted method.^47^ It measures PA in 3 axes and intensity (sedentary, light, moderate or vigorous). A minimum of 5 consecutive days of monitoring is required for reliably estimating PA from Actigraph data in adults.^48^ For each willing participant, one wrist accelerometer was sent by mail to their preferred address between June and October 2023. The participants were advised to wear it on their non-dominant wrist for 7 consecutive days on a normal week for them, 24 hours a day, starting at any time of their choosing. This approach ensured that a full 7 days of measurement would be recorded, including 1 weekend (Saturday and Sunday). All participants were instructed to wear the accelerometer including during sleep, except when bathing, showering or swimming, ensuring consistency and accuracy across participants. The device was fully charged when delivered to participants, so they did not need to charge it during the 7-day period. Following 7 days of wear, participants were asked to return their device by mail using an envelope with postage prepaid.

The device was initialised at a frequency of 30 Hz and accelerometer data were downloaded using ActiLife software V.6.6.3 and were analysed in 60 s epochs. For the analysis in ActiLife software, data were considered valid only if the participant used the accelerometer for a minimum of 10 hours of daily recordings and at least 4 days including a weekend day.^49^ Periods with consecutive values of zero (with a 2 min spike tolerance) for 60 min or longer were interpreted as ‘accelerometer not worn’ and were excluded from the analysis.^50^ Night-time sleep was excluded by removing the hours between 00:00 and 06:00 on each day and potential daytime sleep was considered as sedentary time. The time spent in each intensity of PA and sedentary time were estimated based on the cut points proposed by Freedson *et al*,^51^ considering sedentary time as 0 to 99 counts per minute (cpm), light PA as 100 to 1951 cpm, and moderate-to-vigorous PA (MVPA) as ≥1952 cpm using the vertical axis and analysed in minutes/week, adjusting for the number of days and daily hours that the device was worn. Although waist-worn accelerometers have been more commonly used to assess PA, wrist-worn accelerometers were specifically chosen to maximise wear compliance.^52^ For the analysis in this study, the percentage of MVPA was used as an outcome measure for the device-based PA, which was automatically calculated by ActiLife software based on the cut point algorithm and criteria, as explained above.

#### Activity fluctuations

Based on the cut point algorithm and criteria we used, the ActiLife software also automatically calculated the cpm for each minute of each day of the monitored data. Activity fluctuations or activity variability has been referenced in the literature as an accelerometer-derived measure of an aspect of activity pacing.^13 53^ Therefore, to calculate the activity fluctuations, the SD of cpm was first computed for each individual hour using the vector magnitude, a variable that combined the 3 axes in one outcome defined as √(x² + y² + z²). This was calculated across three time periods during the day (morning: 06:00–12:00; afternoon: 12:00–18:00; evening: 18:00–12:00), producing 6 hourly SD values per time period per day. These 6 hourly SD values were then averaged to produce a single activity fluctuation value for each time period per day. Finally, these daily values were averaged across the 7 monitored days (Monday–Sunday) to produce a weekly average for each time period. The measure of activity fluctuations during an average day in the week provides insight into the distribution of activities over the day, which contributes to characterising an important element of activity pacing behaviour.

### Covariates determination

Referring to previous literature, relevant covariates were considered as potential confounding factors in our analysis,^28 54^ including sociodemographic characteristics (age, biological sex), lifestyle factors (body mass index, BMI), chronic disease conditions (duration of condition, pacing groups (if they have received fatigue management advice) and years of fatigue management advice if received). Ages were recorded as whole years and analysed as continuous values without grouping into categories. Biological sex was dichotomised into male and female. BMI was calculated as body mass in kilograms divided by metres squared and categorised as<25, 25–29 and ≥30. Duration of condition was categorised as 3–6 months, 6–12 months, 1–2 years and over 2 years. The covariate ‘pacing groups’ was dichotomised into the advice group (‘received fatigue management advice’) and the non-advice group (‘not received fatigue management advice’). For the participants in the advice group, the variable ‘years of fatigue management advice’ was categorised into 1–6 months, 6–12 months, 1–5 years and 5–10 years. For participants in the non-advice group, a value of zero was assigned.

### Statistical analyses

Statistical Package for the Social Science V.28 (IBM SPSS Statistics)^55^ was used for the statistical analyses. The study participants were described with descriptive statistics. The normality assumption for continuous measures was tested using the Shapiro-Wilk test. Normally distributed continuous variables were reported with mean (M) and SD and independent T-test was used to compare the two groups. Non-normally distributed continuous variables were reported using median (Mdn), and IQR and Mann-Whitney U test was used to compare the two groups. Frequency (n) and percentage (%) were used for the categorical variables and Fisher’s exact test was used to compare the two groups. The significance level was set at a p<0.05 for the study’s statistical analyses. Results of the mixed model regression analyses were reported as standardised regression coefficients (β) with 95% CIs.

#### Aim 1: activity fluctuations differences between advice versus non-advice group

Univariable mixed model: To investigate if activity fluctuation is different between adults who have received fatigue management advice versus those who have not, we performed one univariable mixed model analysis with average activity fluctuation in the morning, afternoon, and evening over all days (element of activity pacing) as the dependent variable and pacing groups as the independent variable. Random intercepts at participant level were added. The normality of the residuals was checked by performing linear regression analyses.

Multivariable mixed model: Second, the above model was corrected for biological sex, age, BMI, duration of condition, and years of fatigue management advice.

Third, we also corrected for the fatigue score in addition to the variables mentioned above, due to the imbalance between the groups regarding this variable (biological sex, age, BMI, duration of condition, and years of fatigue management advice and fatigue).

#### Aim 2: activity fluctuations associated with fatigue, pacing, HRQoL, self-regulation of PA and PA

To examine whether activity fluctuation was associated with fatigue, engagement in pacing, perceived risk of overactivity, HRQoL, self-regulation of PA, and PA (self-reported and device-based) among all the participants, we performed seven multivariable mixed models with average activity fluctuation (a measurable aspect of activity pacing) in the morning, afternoon, and evening as the dependent variable and each of the seven variables as an independent variable: fatigue, perceived engagement in activity pacing, perceived risk of overactivity, HRQoL, self-regulation of PA, self-reported PA, and device-based PA. Random intercepts at participant level were added. The residuals of the linear regression model with fatigue and self-reported PA associated with average SD of cpm were non-normally distributed; therefore, we log-transformed fatigue and self-reported PA and used the log-transformed fatigue and self-reported PA variables in the analyses. We also checked for independence of observations, linearity between the independent and dependent variables, and homoscedasticity, and all these assumptions were met. The models were corrected for biological sex, age, BMI, duration of condition, and years of fatigue management advice. Then, the pacing groups were added as effect modifiers in the models.

The linear mixed models have benefits over traditional analysis of variance (ANOVA), as linear mixed models allow the inclusion of both fixed and random effects and can help minimise type-1 error.^56^ Additionally, linear mixed models can help increase statistical power compared with traditional ANOVA and can deal with missing data.^57^

#### Aim 3: distinct pacing patterns

Descriptive analyses: To explore and identify any distinct pacing patterns over the different days of the week for all participants, the activity fluctuation was visualised in figures to illustrate the activity pacing patterns over each day of the week for morning, afternoon, and evening for each participant. All participants were grouped based on visualisation (shape of activity fluctuation and frequency) of their daily activity pacing patterns. To form a pacing pattern group, the same pattern had to be observed on at least 3 out of the 7 days. Descriptive statistics were presented for each of the identified pacing pattern groups. The figures were created in Matplotlib using Python.

As a sensitivity analysis, all models were re-estimated excluding self-reported BMI as a covariate, given the potential for reporting bias in self-reported height and weight.

## Results

A total of 29 participants (82.8% women) with a median age of 54 (IQR of 60) were included in this study, and 96.6% had experienced fatigue for over 2 years. Table 1 shows the participant characteristics of each pacing group (advice group vs non-advice group). The two groups differ on fatigue (p=0.004) and self-reported PA (p=0.053). Participants in the advice group reported a median score of 6.47, indicating significant fatigue. The non-advice group reported significant fatigue with a median score of 4.44. For participants’ specific diagnoses, please see online supplemental table 1.

**Table 1:** Descriptive of participants (N=29)

	All	Received fatigue management advice (advice group)	No fatigue management advice (non-advice group)	P value
Number	29	18	11	
Age (years)	54.0 (60)	54.5 (27.8)	54.0 (34)	0.840
Body mass (kg)	70.0 (22)	75.5 (30.3)	69.8 (15.0)	0.787
Height (cm)	168.2±7.9	168.7±8.2	167.3±7.7	0.528
BMI (kg×m^-2^)	27.4 (5.8)	28.1 (7.5)	26.0 (5.7)	1.000
Valid actigraph days	8.0 (0.5)	8.0 (1.0)	8.0 (0.0)	0.356
Biological sex				0.720
Female	24 (83%)	13 (72%)	11 (100%)	
Male	5 (17%)	5 (28%)		
Employment status				0.791
Full-time	5 (17%)	3 (17%)	2 (18%)	
Part-time	4 (14%)	2 (11%)	2 (18%)	
Not employed	6 (21%)	5 (28%)	1 (9%)	
Retired	11 (38%)	6 (33%)	5 (46%)	
Student	3 (10%)	2 (11%)	1 (9%)	
Education				0.974
Secondary school	2 (7%)	1 (6%)	1 (9%)	
Postsecondary	5 (17%)	3 (17%)	2 (18%)	
Vocation qualification	3 (10%)	2 (11%)	1 (9%)	
Bachelor’s degree	7 (24%)	5 (28%)	2 (18%)	
Postgraduate degree	12 (41%)	7 (39%)	5 (46%)	
Marital status				0.189
Married	12 (41%)	9 (50%)	3 (27%)	
Cohabiting	5 (17%)	1 (6%)	4 (37%)	
Single	6 (21%)	4 (22%)	2 (18%)	
Divorced	6 (21%)	4 (22%)	2 (18%)	
Duration of condition				0.621
1–2 years	1 (3%)	1 (6%)		
Over 2 years	28 (97%)	17 (94%)	11 (100%)	
Length of time fatigue management advice received				N/A
1–6 months		5 (28%)		
6–12 months		2 (11%)		
1–5 years		9 (50%)		
5–10 years		2 (11%)		
Fatigue	6.11 (2.28)	6.47 (1.05)	4.44 (2.77)	0.004*
Engagement in AP	17.69±5.47	18.72±5.14	16.0±5.81	0.408
Perceived risk of overactivity	8 (2)	8 (1.25)	8 (3)	0.927
Self-regulation of PA	33.69±9.09	32.33±10.13	35.91±6.92	0.180
Self-reported PA	2829.48 (2748.02)	2010.39 (1759.33)	4169.82 (3563.55)	0.053
Device-based PA	11.92 (9.88)	13.43 (12.11)	10.31 (4.91)	0.290
HRQoL	56.46±16.14	53.57±14.82	61.20±17.78	0.605
Activity fluctuations	1009.43±407.12	1030.31±389.76	975.26±438.04	0.581

Values presented are M±SD for normally distributed variables, Mdn (IQR) for non-normally distributed variables or N (%) for categorial variables.

* p<0.05.

AP, activity pacing; BMI, body mass index; HRQoL, health-related quality of life; M, mean; Mdn, median; N/A, not available; PA, physical activity.

### Aim 1: activity fluctuations differences between advice versus non-advice group

The univariable mixed model revealed that the two groups did not significantly differ on activity fluctuation during the day (morning, afternoon and evening) (β=−55.04; p=0.654; 95% CI (−303.30 to 193.21)). Similarly, the multivariable model, corrected for confounders, did not reveal a significant difference between the two groups on activity fluctuation (β=−274.16; p=0.245; 95% CI (−746.57 to 198.25)). After adding the fatigue score to the multivariable model adjusted for confounders, the analysis still did not show a significant difference in activity fluctuation between the two groups (β=−383.88; p=0.102; 95% CI (−848.71 to 80.95)).

### Aim 2: activity fluctuations associated with fatigue, pacing, HRQoL, self-regulation of PA and PA

The multivariable mixed model showed that there is a significant positive association between device-based PA and average activity fluctuation during the day for the whole sample (β=47.31; p<0.001; 95% CI (37.08 to 57.54)). No other significant associations were found between activity fluctuation and fatigue, engagement in pacing, perceived risk of overactivity, self-regulation of PA, self-reported PA and HRQoL (p>0.05).

The multivariable mixed models showed that the effects of self-regulation of PA and device-based PA on average activity fluctuation during the day were significantly different between the two groups (respectively, β=1684.14; p=0.006; 95% CI (−68.05 to −12.77) and β=288.83; p=0.042; 95% CI (−45.50 to −0.87)), while corrected for confounders. Calculations are based on β of advice group + (β of the independent variable x pacing group 1) with reference being non-advice group. Results can be found in table 2. No other significant interaction effects were found between average activity fluctuation during the day and fatigue, engagement in pacing, perceived risk of overactivity, self-reported PA, and HRQoL (p>0.05).

**Table 2:** Associations of covariates with activity fluctuation—overall and separate per group

	Model 1 fatigue		Model 2 engagement in pacing		Model 3 perceived risk of overactivity		Model 4 self-regulation of PA		Model 5 self-reported PA		Model 6 device-based PA		Model 7 HRQoL	
	β (95% CI)	P value	β (95% CI)	P value	β (95% CI)	P value	β (95% CI)	P value	β (95% CI)	P value	β (95% CI)	P value	β (95% CI)	P value
Association with activity† fluctuation (without group and interaction)	−300.8 (−759 to 158.1)	0.190	−17.7 (−42.0 to −6.7)	0.149	−22.9 (−103.4 to −57.6)	0.565	9.9 (−4.0 to 23.8)	0.157	129.3 (−54.5 to −313.2)	0.161	47.3 (37.1 to −57.5)	<0.001*	5.0 (−3.3 to −13.2)	0.226
Interaction with group†														
Covariate (ref: non-advice group)	−176.6 (−710.3 to 357.7)	0.504	−11.8 (−43.2 to 19.6)	0.449	5.15 (−91.2 to 101.5)	0.914	41.1 (17.6 to 64.5)	0.001*	59.8 (−242.4 to 361.9)	0.688	62.9 (44.9 to 81.0)	<0.001*	12.4 (1.7 to 23.2)	0.025*
Advice group	1568.3 (−108.4 to 3245.0)	0.066	525.4 (−367.1 to 1417.8)	0.238	697.9 (−533.1 to 1928.9)	0.256	1726.6 (695.6 to 2757.4)	0.002*	−153.9 (−2416.6 to 2108.8)	0.890	312.0 (−95.2 to 719.6)	0.132	1064.1 (104.5 to 2023.7)	0.031*
Covariate*Advice group	−740.7 (−1751.3 to 269.8)	0.145	−14.7 (−62.0 to 32.7)	0.532	−63.25 (−227.2 to 100.7)	0.436	−40.41 (−68.1 to −12.8)	0.006*	114.89 (−506.88 to 736.66)	0.708	−23.2 (-45.5 to −0.9)	0.042*	−12.4 (−26.7 to 2.0)	0.089

* p<0.05.

† Models were corrected for biological sex, age, BMI, duration of condition and years of fatigue management advice.

BMI, body mass index; HRQoL, health-related quality of life; PA, physical activity; ref, reference category; β, standardised regression coefficient.

### Aim 3: distinct pacing patterns

**Figure 1:**
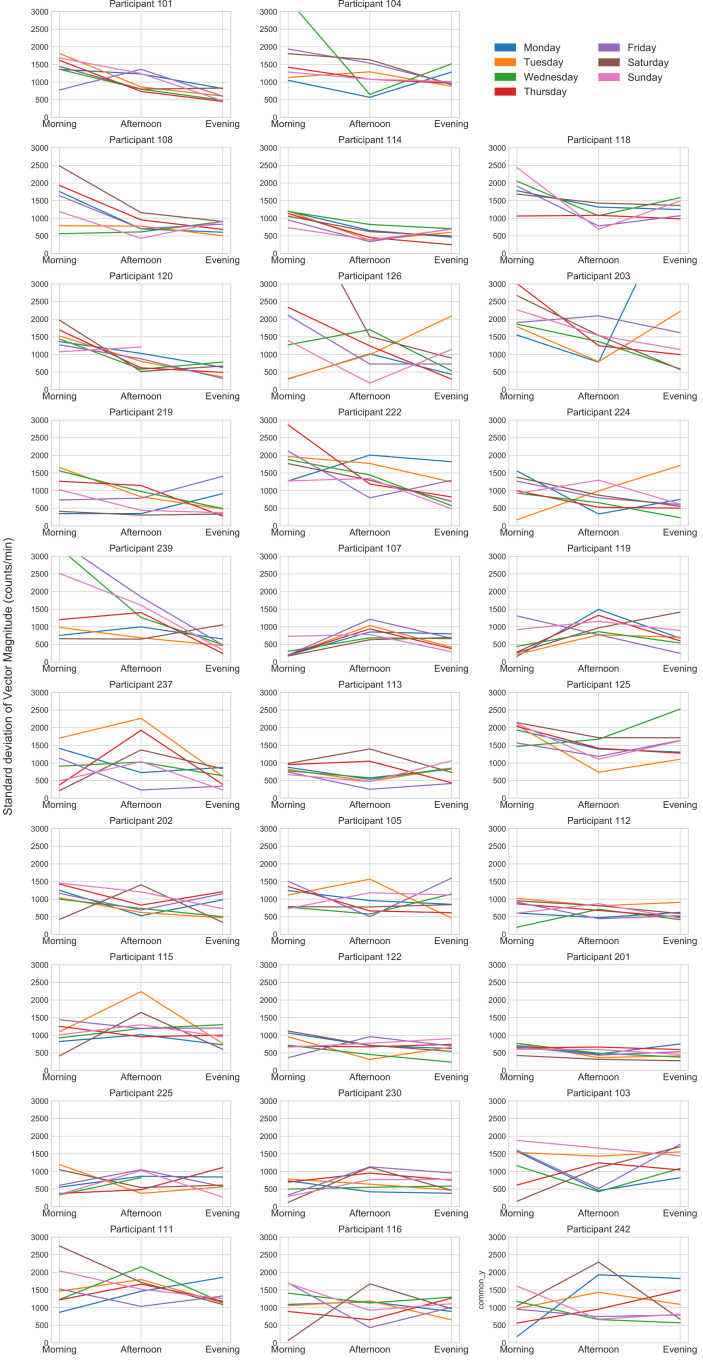
Pacing patterns among participants.

The different pacing patterns among all participants are illustrated in figure 1. The number (no) of participants starting with ‘1’ indicates those belonging to the advice group, while those starting with ‘2’ indicate participants from the non-advice group. Through the visualisation of individual patterns, five distinct activity pacing patterns were identified: (1) high fluctuations in the morning, (2) high fluctuations in the afternoon, (3) high fluctuations at two time points (morning and evening), (4) consistent pacing pattern and (5) varied pacing patterns. Therefore, all participants were assigned to one of the five pacing subgroups based on the pacing patterns explained above. Among the 29 participants, 12 (participant no.: 101, 104, 108, 114, 118, 120, 126, 203, 219, 222, 224, 239) exhibited high fluctuations in the morning with SD peaking during the morning hours but decreasing thereafter. A smaller group of three participants (participant no.: 107, 119, 237) was allocated to the high fluctuations in the afternoon subgroup with SD peaking in the afternoon but remaining at a lower level during morning and evening hours. Three participants (participant no.:113, 125, 202) demonstrated high fluctuations at two time points per day, with peaking both in the morning and evening while remaining low in the afternoon. Seven participants in the consistent pacing pattern subgroup (participant no.: 105, 112, 115, 122, 201, 225, 230) accumulated moderate levels of SD throughout the day and the week. The varied pacing patterns subgroup with four participants (participant no.: 103, 111, 116, 242) showed the highest fluctuations of diverse patterns observed throughout the week.

The descriptives of participants in each subgroup based on the observed groups above can be found in table 3. No statistical test was conducted to compare these subgroups due to the small sample sizes. On visually examining the characteristics (mean±SD or (Mdn(IQR)) of participants within each subgroup, subgroup 1 has the lowest fatigue score (5.16 (2.48)), but they also exhibit the lowest engagement in pacing (15.08±6.12). Additionally, they demonstrate a high score in self-regulation of PA (35.17±10.94), device-based PA (14.93±5.91), and the highest score in HRQoL (63.63±15.54). In contrast, subgroup 2 shows the highest fatigue score (6.6 (0)) and the highest score in self-reported PA (5315.0 (7104.2)), along with the lowest score in device-based PA (6.96±3.02). Subgroup 3 exhibits the highest scores in self-regulation of PA (40.67±2.31) and in engagement in pacing (22.33±1.53). Subgroup 4 displays the lowest scores in HRQoL (47.91±17.99) and perceived risk of overactivity (7 (2)) with a high score in self-regulation of PA (32.43±8.54). Lastly, subgroup 5 demonstrates the highest score in perceived risk of overactivity (8.5 (2.5)) and device-based PA (17.52±4.33), along with the lowest score in self-reported PA (1519.5 (1388.1)) and a high score in HRQoL (61.19±17.89).

**Table 3:** Descriptive of participants based on five pacing subgroups

	1	2	3	4	5
Number	12	3	3	7	4
Age (years)	56.5 (19.75)	62.0 (0)	35.0 (0)	52.0 (32)	54.0 (18)
Body mass (kg)	72.5 (25)	78.0 (0)	81.0 (0)	76.0 (45)	64.0 (13.3)
Height (cm)	169.7±7.5	167.0±14.2	167.0±3.0	167.6±8.4	166.5±9.3 5±9.3
BMI (kg×m^-2^)	27.8 (5.6)	29.7 (0)	28.0 (0)	29.0 (16.1)	22.4 (1.2)
Biological sex					
Female	9 (75%)	2 (66%)	3 (100%)	7 (100%)	3 (75%)
Male	3 (25%)	1 (33%)			1 (25%)
Pacing group					
Group 1	7 (58)	2 (67%)	2 (67%)	4 (57)	3 (75%)
Group 2	5 (42)	1 (33%)	1 (33%)	3 (43)	1 (25%)
Employment status					
Full-time	4 (33%)	–	–	–	1 (25%)
Part-time	2 (17%)	–	–	–	2 (50%)
Not employed	2 (17%)	1 (33%)	2 (67%)	1 (14%)	–
Retired	4 (33%)	2 (67%)	1 (33%)	3 (43%)	1 (25%)
Student	–	–	–	3 (44%)	–
Education					
Secondary school	1 (8%)	1 (33%)	–	1 (9%)	–
Sixth form college	1 (8%)	1 (33%)	1 (33%)	1 (14%)	–
Vocation qualification	–	–	2 (67%)	1 (14%)	1 (25%)
Undergraduate university	4 (33%)	–	–	1 (14%)	2 (50%)
Postgraduate	16 (60%)	1 (33%)	–	4 (57%)	1 (25%)
Marital status					
Married	5 (42%)	2 (67%)	1 (33%)	1 (14%)	3 (75%)
Cohabiting	4 (33%)	–	–	1 (14%)	–
Single	2 (17%)	1 (33%)	1 (33%)	2 (29%)	–
Divorced	1 (8%)	–	1 (33%)	3 (43%)	1 (25%)
Duration of condition					
1–2 years	–	–	1 (33%)		
Over 2 years	12 (100%)	3 (100%)	2 (67%)	7 (100%)	4 (100%)
Fatigue	5.16 (2.48)	6.6 (0)	5.88 (0)	6.22 (3.66)	6.05 (2.84)
Engagement in activity pacing	15.08±6.12	20.67±2.52	22.33±1.53	18.57±5.71	18.25±3.30
Perceived risk of overactivity	8 (2)	8 (0)	8 (0)	7 (2)	8.5 (2.5)
Self-regulation of PA	35.17±10.94	30.33±5.86	40.67±2.31	32.43±8.54	28.75±7.36
Self-reported PA	2989.2 (1813.8)	5315.0 (7104.2)	2039.0 (2019.2)	2577.8 (2322.2)	1519.5 (1388.1)
Device-based PA	14.93±5.91	6.96±3.02	12.75±10.33	9.26±5.17	17.52±4.33
HRQoL	63.63±15.54	49.39±5.43	48.50±7.57	47.91±17.99	61.19±17.89

Values presented are M±SD for normally distributed variables, Mdn (IQR) for non-normally distributed variables or N (%) for categorial variables;

1, high fluctuations in the morning; 2, high fluctuations in the afternoon; 3, high fluctuations at two time points (morning and evening); 4, consistent pacing pattern; 5, varied pacing patterns.

BMI, body mass index; HRQoL, health-related quality of life; M, mean; Med, median; PA, physical activity.

Results from the sensitivity analyses excluding self-reported BMI are presented in online supplemental table 2. These analyses yielded results that were consistent with the primary analyses, with no changes observed in the direction or statistical significance of the main or interaction effects.

## Discussion

In this cross-sectional study, while no significant differences in activity fluctuations were found between individuals with chronic conditions who received fatigue management advice and those who did not, our findings highlight key associations. We found a positive relationship between activity fluctuations and device-based PA across the entire sample, suggesting that greater fluctuations may be characteristic of certain PA patterns. Additionally, self-regulation and device-based PA associated with daily activity fluctuations showed significant interaction effects between the groups, indicating distinct pacing patterns emerging. These findings provide important insights into how individual differences in activity regulation and behaviour may inform the personalisation of fatigue management strategies, particularly by identifying varying pacing styles and their potential impact on PA engagement.

Two major findings emerged from the analyses for the second and third aims. For the second aim, we found that the positive association between activity fluctuations and self-regulation of PA was significantly stronger in the advice group than in those in the non-advice group. This finding may indicate that individuals who receive advice might know how to better manage their activity levels and take a more goal-directed approach (as previously has been reported by individuals with chronic conditions.^4 5^ A review on fatigue management for adults with multiple sclerosis found strong evidence that group fatigue management education is effective, suggesting that receiving structured advice and education can help individuals become more aware of their limitations and manage their activity levels effectively.^58^ Although activity fluctuations did not differ between individuals who did and did not receive fatigue management advice (in aim 1), the advice might strengthen the association between self-regulation of PA and activity fluctuations. This suggests that fatigue management support may enhance individuals’ ability to regulate their activity patterns in a more goal-directed manner.^6^ Consequently, they might engage in a pattern involving rest periods to manage their fatigue symptoms and intense PA, leading to greater activity fluctuation. Insufficient and excessive rest have been shown to negatively affect physical and mental health, potentially contributing to increased disability or burnout.^59^ This dynamic highlights the need for a balanced approach, where rest and activity are strategically linked to promote optimal well-being, while ensuring that rest is operationalised and measured effectively in relation to the specific needs of individuals with chronic conditions and fatigue.^60^ Notably, activity fluctuation is an element of activity pacing. Thus, consistent-without many fluctuations, could also indicate pacing to avoid intense PA levels in anticipation to fatigue.^61^ However, it is important to note that measuring fluctuations in our study captures activity, not rest or the quality of rest, and an increase in fluctuation can also indicate an increase in rest periods.

Furthermore, a higher percentage of MVPA was significantly positively associated with activity fluctuation. However, this relationship is interdependent, as activity fluctuation is derived from device-based PA, and could be influenced by factors such as rest, activity intensity and self-regulation.^59^ Pilot studies have shown promising effects of activity pacing interventions in enhancing PA behaviour.^2 12 62^ These interventions often emphasise the importance of balancing activity with rest to prevent overexertion, which could help explain the fluctuations observed in our study.^60^ Our finding suggests that individuals who engage in more MVPA might exhibit higher activity fluctuation throughout the day. This makes interpretation of this finding challenging and could have two potential explanations. First, these individuals might be taking more frequent rest breaks or engaging in lower intensity activities as well, leading to more fluctuations. Rest breaks are recognised as an effective way to manage fatigue and maintain performance^6 63 64^; however research on rest in chronic conditions is still scarce, particularly in relation to PA behaviour. Second, individuals might struggle with self-regulating their activities, not knowing when to stop^4^ which could lead to overactivity and boom-and-bust cycles.^65^ Boom-and-bust cycles, characterised by periods of excessive activity followed by prolonged rest,^66^ can contribute to higher fluctuations.^3^ The key lies in self-regulation: preplanned alternation of activity and rest is beneficial, whereas unplanned bursts followed by prolonged rest contribute to less effective fatigue management. Future research exploring the interplay between rest, activity regulation, and fatigue is essential,^6^ as fatigue management programmes integrating self-regulation strategies may help individuals better balance activity and rest.^3 59^ However, our current measure of activity fluctuations does not capture rest specifically, which remains a limitation requiring further exploration.

Furthermore, a positive significant interaction effect was found, indicating that the relationship between device-based PA and activity fluctuations differed depending on whether someone received fatigue management advice, with a stronger relationship observed in the advice group. This suggests that fatigue management advice may encourage individuals to engage in more PA of varying intensity throughout the day, leading to fluctuations, potentially enabled through more frequent rest breaks. Such fluctuations could indicate a more adaptive pacing strategy,^67^ where individuals balance periods of activity with rest to optimise energy levels and manage fatigue effectively.^60^

For aim 1, no significant differences in activity fluctuations were found between groups, which may be due to the small sample size and the variability in duration of advice received (ranging from 1 to 6 months to 5–10 years). Learning to pace activities effectively is a long-term developmental process, as evidenced in both sports and healthcare contexts.^68^ Furthermore, the advice group had significantly higher fatigue (FSS: 6.47 (1.05)) compared with the non-advice group (4.44 (2.77)), though both groups scored above the clinical threshold of 4.0.^39 40^ This could indicate that more severe cases are more frequently referred to fatigue management services, suggesting that preventive advice for individuals with early fatigue symptoms may be beneficial before symptoms worsen.

The above findings highlight the importance of tailored fatigue management advice that considers an individual’s baseline, goals and needs.^6 69^ The non-advice group consisted entirely of females, while the advice group included 72% females, possibly reflecting the higher prevalence of fatigue-related conditions among women.^70^ The difference in fatigue between the two groups was statistically significant, with the higher score indicating more severe fatigue, affecting the person’s activities to a greater extent. Moreover, the two groups significantly differed in self-reported PA, with advice group reporting lower self-reported PA compared with non-advice group. This difference may also be explained by the significant difference in fatigue severity, as the non-advice group reported less fatigue (but still significant above total score of 4.0 in FSS) but reported to be engaged in higher levels of PA METS over the week.

Moreover, in aim 3, we identified and described five distinct pacing patterns among adults with chronic conditions. Subgroup 1, characterised by high morning fluctuations, reported the lowest fatigue, lowest perceived engagement in pacing, highest device-based PA and highest HRQoL. This aligns with previous findings that decreased engagement in pacing is associated with increased HRQoL,^4^ suggesting a greater need for pacing when fatigue levels are high and quality of life is impacted. Subgroup 2, characterised by high afternoon fluctuations, reported the highest fatigue and self-reported PA but the lowest device-based PA. This discrepancy between perceived and objective activity levels underscores the limitations of self-reported PA^71 72^ and may contribute to their higher fatigue levels, while also highlighting the added value of combining both measures. Subgroup 3, characterised by high fluctuations in both morning and evening, possibly suggesting anticipated rest in between, reported the highest self-regulation of PA and engagement in pacing but low HRQoL. This implies that individuals who consciously engage more in pacing may experience decreased HRQoL,^4^ and that increased self-regulation and pacing engagement did not appear to result in reduced fatigue or increased PA.

Subgroup 4, characterised by a consistent pacing pattern, reported the lowest HRQoL and perceived risk of overactivity, alongside high self-regulation of PA. Despite increased self-regulation, the decreased HRQoL may indicate that while their consistent pattern prevents overactivity, high fatigue levels still severely impact daily life, potentially leading to avoidance of active bouts of PA in anticipation of fatigue.^61^ Subgroup 5, characterised by varied pacing patterns during the week, reported the highest perceived risk of overactivity and device-based PA, the lowest self-reported PA and self-regulation, and high HRQoL. Despite perceiving overactivity risk, these individuals engage in high device-measured PA but may underestimate their activity levels when self-reporting. The high HRQoL could be attributed to their increased PA levels.^73^ Notably, 75% of this subgroup belonged to the advice group; however, all five pacing patterns included individuals from both groups, suggesting that some patterns may be followed regardless of advice received.

Andrews *et al* conducted a systematic review on activity patterns in chronic pain with activity pacing, avoidance and endurance identified as the three main activity patterns in chronic pain.^74^ Birkholtz and Aylwin found that in chronic pain, pacing involves various behavioural strategies, such as frequent short breaks, dividing tasks into manageable segments, frequently alternating positions and tasks and slowing down.^75^ Fordyce identified two types of maladaptive activity pacing patterns in chronic pain: individuals who rest when pain is greater and individuals who were unable to stop activity.^65^ Although these studies focus on chronic pain, they may relate to fatigue, as both symptoms often overlap in coping with the symptoms and impact on daily life.^76^ Abonie and Hettinga conducted a study on a tailored activity pacing intervention in multiple sclerosis, which identified two key activity profiles before intervention start: individuals with activity avoidance and those with overactive behaviour followed by prolonged rest.^2^ The intervention aimed to balance PA and fatigue management by tailoring pacing strategies to individual behaviours and these two distinct patterns that were discussed with participants. Findings showed that tailored pacing improved PA levels and reduced variability without exacerbating fatigue.^2^ While this study focused on two distinct profiles based on findings in a meta-analysis,^14^ our findings expand on this by identifying five distinct pacing patterns. These patterns suggest that, in chronic conditions, activity pacing guidance is even more complex and should be further tailored and individualised based on additional characteristics in addition to avoidance and overactivity behaviours. Thus, our novel descriptive findings provide insights into how individuals pace throughout the week.

### Implications

The overall findings from this study highlight the complexity and potential for tailoring activity pacing guidance based on activity fluctuation patterns and other variables in fatigue management among chronic conditions. The non-insignificant findings for the first aim, examining whether activity fluctuations over a period of 1 week differ between individuals who had and had not received fatigue management advice, could indicate that individuals with higher levels of fatigue (eg, advice group) might be referred to fatigue management services more frequently than others. This reactive approach may result in individuals with less severe fatigue missing out on timely management advice,^77^ potentially allowing their fatigue symptoms to worsen over time before they finally receive the necessary support. This could indicate a shift toward curative care rather than proactive fatigue management, which may ultimately perpetuate a cycle of worsening symptoms and increased treatment burden. The higher fatigue severity in the advice group that received fatigue management advice compared with the non-advice group could be important for future research on the complex interplay between fatigue severity, advice effectiveness, and activity patterns and behaviours. Balancing PA and rest are important factors to manage daily life activity engagement in adults who experience fatigue.^6 30^ For some individuals, the lack of awareness of when to stop can lead to overactivity, especially when coupled with prolonged rest periods, contributing to boom-and-bust cycles.^4^ Our findings suggest that activity patterns and behavioural characteristics vary across individuals, highlighting the need for tailored approaches to prevent extreme fluctuations in activity levels.^3 4 15^ The question now is how clinicians might be able to understand different profiles and work with the individuals to give tailored support. The present study provides a potential approach that can be used to personalise advice and treatment, to be used on lifestyle interventions aiming to promote a healthy lifestyle such as those described in ReSpAct.^77^,^79^ Further research on this topic is required. The distinct pacing patterns identified provide a nuanced understanding of how individuals with chronic conditions pace their activities. A tailored approach might consider individual needs, goals, and baseline levels of fatigue, PA and self-regulation.^6^ Additionally, these findings underscore the importance of personalised interventions in clinical practice, which can offer user satisfaction and promote healthy behaviours^80 81^ and provide tailored treatments and preventions strategies.^82^ By acknowledging fluctuations among individuals in healthcare, clinicians can better support individuals managing their conditions, potentially improving their overall HRQoL.^4 27^

### Strengths and limitations

While cross-sectional analyses can have limitations in observational research, our use of mixed-model analysis represents an initial exploration into this under-researched are of pacing patterns in individuals with chronic conditions. The inclusion of participants who have received fatigue management advice from existing practices provides valuable insights into current fatigue management and activity pacing behaviours of people living with chronic conditions. The novel findings of this study might enhance the understanding of the distinct pacing patterns and can inform the discussions of healthcare professionals along with tailored intervention development. The inclusion of a diverse sample of participants from different chronic conditions-transdiagnostic approach- enriches the findings, providing a broader perspective on pacing behaviours across various conditions, which can help tailor interventions to specific needs. Also, based on these findings and methodology, we might be able to profile individuals with chronic conditions on pacing behaviours. This profiling could aid in the development of personalised approaches to fatigue management, making interventions more targeted and effective. Lastly, the combination of self-reported data, device-based PA measurements and psychological factors like self-regulation is a first step towards a holistic view of how individuals with chronic conditions manage their activity.

Limitations of this research should also be noted. First, self-reported measurements of fatigue, engagement in pacing, perceived risk of overactivity, self-regulation of PA, self-reported PA and HRQoL could be affected by memory bias as participants were required to recall experienced over the previous 7 days. Second, drug use information was not collected from participants; however, including it as a confounder in future studies would be important. Third, although this study included the independent variables of fatigue, engagement in pacing, perceived risk of overactivity, self-regulation, PA and HRQoL, other variables may play a role in influencing activity fluctuations including the build environment and rest^6^ ; thus, further research should consider the inclusion of other variables. Fourth, this study was conducted only in one part of the UK. Moreover, another limitation is that the relationship between MVPA and activity fluctuations is interdependent, and this finding should be interpreted with this in mind. Also the cross-sectional nature of this study along with the im balance between the groups restrict our ability to determine causation. Further research with larger sample sizes should focus on longitudinal and intervention studies as it can allow for an in-depth exploration of activity pacing patterns and explore these relationships and fatigue management advice further. Furthermore, while the ActiGraph wGT3X-BT is a validated device for assessing PA, the method used to calculate activity fluctuations has not been validated, and further research is needed to establish standardised approaches for quantifying activity variability using accelerometer-derived data. Lastly, while a 7-day accelerometry monitoring period is widely accepted for capturing PA patterns, it may not sufficiently capture activity fluctuations, such as those associated with postexertional malaise episodes, and future research should explore longer monitoring durations.

## Conclusions

This cross-sectional study found no differences in activity fluctuations between adults with chronic conditions who had received fatigue management advice and those who had not. Moreover, the positive associations between activity fluctuation and both self-regulation of PA and device-based PA were significantly different between the two groups. The positive interaction suggests that self-regulation of PA has a stronger impact on activity fluctuation for those receiving fatigue management advice, possibly making them more aware of their limitations and how to manage activity levels. Additionally, the association between device-based PA and activity fluctuation was stronger for those who received fatigue management advice, suggesting that advice may encourage greater fluctuations in PA levels, including frequent rest breaks. Five distinct pacing patterns were descriptively identified among the participants: (1) high fluctuations in the morning, (2) high fluctuations in the afternoon, (3) high fluctuations at two time points (morning and evening), (4) consistent pacing pattern and (5) varied pacing patterns. These findings underscore the importance of a tailored fatigue management approach and suggest that a strictly consistent pacing pattern is not necessarily optimal for everyone. Instead, periods of pre-planned rest might contribute to overall PA engagement and the key is to prevent boom-and-bust cycles and extreme fluctuations in activity levels. Therefore, clinicians should consider the baseline, needs and goals of individuals when designing an effective and personalised strategy. Future research with larger sample sizes and longitudinal designs is needed to further explore these relationships and the multidimensionality of pacing.

## Supplementary material

10.1136/bmjopen-2025-104566 online supplemental file 1

10.1136/bmjopen-2025-104566 online supplemental table 1

10.1136/bmjopen-2025-104566 online supplemental table 2

## Data Availability

Data supporting this study are not available due to privacy and ethical restrictions, as they contain sensitive information that could compromise participant confidentiality.
