# Supplementary material for: Unravelling the diversity of pacing behaviours in adults with chronic conditions: a cross-sectional study
Source: BMJ Open. 2026 May 27;16(5):e104566. doi: 10.1136/bmjopen-2025-104566 (PMC13218175; doi:10.1136/bmjopen-2025-104566)
Supplement: online supplemental table 1 [file bmjopen-16-5-s002.docx]

Supplementary Table 1

Supplementary Table 1. Specific Diseases of Participants

|  | | All participants (N=29) | Group 1  (N=18) | Group 2  (N=11) |
| --- | --- | --- | --- | --- |
| Comorbidity* | 17 (58.6%) | | 12 (66.7%) | 5 (45.5%) |
| Antiphospholipid syndrome | 1 (3.4%) | | 1 (5.6%) |  |
| Aplastic Anemia | 1 (3.4%) | |  | 1 (9.1%) |
| Back pain | 1 (3.4%) | |  | 1 (9.1%) |
| Bronchiectasis | 1 (3.4%) | | 1 (5.6%) |  |
| ME/CFS | 2 (6.9%) | | 2 (11.1%) |  |
| Fibromyalgia | 1 (3.4%) | |  | 1 (9.1%) |
| Long Covid | 1 (3.4%) | | 1 (5.6%) |  |
| Multiple sclerosis | 2 (6.9%) | | 1 (5.6%) | 1 (9.1%) |
| Rheumatoid Arthritis | 1 (3.4%) | |  | 1 (9.1%) |
| Sjogren's Syndrome | 1 (3.4%) | |  | 1 (9.1%) |

Values presented are N (%); N, number; ME/CFS, myalgic encephalomyelitis/chronic fatigue syndrome; *Comorbidities (two or more conditions in combination) include combinations of the following: fibromyalgia, postural orthostatic tachycardia syndrome, chronic vertiginous migraine, myalgic encephalomyelitis/chronic fatigue syndrome, type 2 diabetes, hypertension, depression, fatty liver disease, osteoarthritis, autonomic failure, asthma, restless legs syndrome, functional neurological disorder, irritable bowel syndrome, gastroesophageal reflux disease, hypermobile spectrum disorder, postural tachycardia syndrome, Ehlers-danlos syndrome, chronic costochondritis, Raynaud’s syndrome, temporomandibular disorder, chronic gastric volvulus, Sjögren's syndrome, blood clotting disorder, hypermobility spectrum disorder
