# Supplementary material for: Unravelling the diversity of pacing behaviours in adults with chronic conditions: a cross-sectional study
Source: BMJ Open. 2026 May 27;16(5):e104566. doi: 10.1136/bmjopen-2025-104566 (PMC13218175; doi:10.1136/bmjopen-2025-104566)
Supplement: online supplemental table 2 [file bmjopen-16-5-s003.docx]

Sensitivity analyses without self-reported body mass index (BMI)

Supplementary Table 2. Sensitivity analyses: Associations of Covariates with Activity Fluctuation - Overall and Separate per Group

|  | Model 1  Fatigue | Model 2 Engagement in Pacing | | | Model 3 Perceived Risk of Overactivity | | Model 4 Self-regulation of PA | | Model 5  Self-reported PA | | Model 6  Device-based PA | | Model 7  HRQoL | |  |
| --- | --- | --- | --- | --- | --- | --- | --- | --- | --- | --- | --- | --- | --- | --- | --- |
|  | β  (95%CI) | p | β  (95%CI) | p | β  (95%CI) | p | β  (95%CI) | p | β  (95%CI) | p | β  (95%CI) | p | β  (95%CI) | p | |
| **Association with Activity Fluctuation** (without group and interaction) | -351.5  (-801.6, 98.7) | .121 | -14.4 (-39.3, -10.5) | .247 | -11.9 (-93.1, 69.2) | .766 | 10.0 (-4.3, 24.3) | .163 | 149.8  (-29.6, 329.1) | .098 | 46.6  (36.8, 56.4) | <.001* | 6.0 (-1.4, 13.4) | .107 |  |
|  |  |  |  |  |  |  |  |  |  |  |  |  |  |  |  |
| **Interaction with group** |  |  |  |  |  |  |  |  |  |  |  |  |  |  |  |
| Covariate (ref: non-advice group) | -260.8  (-789.1, 267.5) | .321 | -11.85  (-44.6, 21.6) | .483 | 7.4  (-93.1,  107.8) | .882 | 43.1  (19.7, 66.6) | .001* | 66.7  (-212.7, 346.1) | .629 | 61.7  (44.1, 79.3) | <.001* | 13.5  (2.9,  24.1) | .015* |  |
| Advice Group | 1434.1  (-269.4, 3137.5) | .096 | 355.1  (-563.6, 1273.9) | .436 | 478.5  (-771.2,  1728.1) | .440 | 1804.9 (769.6, 2840.3) | .001* | -172.3  (-1331.1, 986.4) | .763 | 321.0  (-86.5, 728.4) | .121 | 1019.7  (53.7, 1985.8) | .039* |  |
| Covariate*Advice group | -662.5  (-1689.8, 364.8) | .197 | -6.8  (-55.9, 42.3) | .779 | -35.2  (-202.0,  131.5) | .669 | -43.3  (-70.7, -15.8) | .003* | 124.3  (-248.7, 497.3) | .501 | -22.8  (-45.2,  -0.5) | .045* | -11.7  (-26.2,  2.8) | .109 |  |

*p<0.05; **Models were corrected for biological sex, age, duration of condition, and years of fatigue management advice; PA, physical activity; HRQoL, health-related quality of life; β, standardized regression coefficient; 95%CI, 95% confidence interval; ref, reference category
