# Supplementary material for: Unravelling the diversity of pacing behaviours in adults with chronic conditions: a cross-sectional study
Source: BMJ Open. 2026 May 27;16(5):e104566. doi: 10.1136/bmjopen-2025-104566 (PMC13218175; doi:10.1136/bmjopen-2025-104566)
Supplement: Supplementary file 4 [file bmjopen-2025-104566.reviewer_comments.pdf]

PEER REVIEW HISTORY

BMJ Open publishes all reviews undertaken for accepted manuscripts. Reviewers are asked to complete a checklist review form and are provided with free text boxes to elaborate on their assessment. These free text comments are reproduced below.

ARTICLE DETAILS

Title (Provisional)

Unravelling the Diversity of Pacing Behaviours in Adults with Chronic Conditions: A Cross-Sectional Study

Authors

Barakou, Ioulia; Seves, Bregje L.; Abonie, Ulric; Finch, Tracy; Hackett, Katie; Hettinga, Florentina

---

VERSION 1 - REVIEW

**Reviewer**	**1**
**Name**	**Gray, Emily**
**Affiliation**	**University of Otago, School of Physiotherapy**
**Date**	**10-Jul-2025**
**COI**	**None**

The manuscript is well written. The background information is clear, relevant and justifies the aim of the study. The aims are clearly stated. The methods are mostly thorough, with established outcome measures utilized relevant to the study aims. There are several areas related to participant recruitment and eligibility that need further explanation/clarification (see comments below). The results are clearly documented with good use of tables and figures. The discussion is in-depth and appropriately draws on relevant literature. The study strengths and limitations are appropriately described (with potentially another couple of limitations to include depending on responses to my comments/queries below). The conclusions are appropriate and does not overstate the study findings.

Comments:

1. Please provide more information about how the potential participants from the fatigue clinic were invited by clinicians. Were all eligible participants invited? Or were only some selected to invite? If so, how were these people selected? How many people were invited in total? If there is potential for selection bias this needs to be included in the study limitations.

2. Was there any data collected regarding how long ago the participants had received the fatigue advice? If it was a long time ago this could affect implementation of the advice if participants forget key information over time.

3. The study sample included adults with chronic conditions who experience fatigue. Some participants were recruited from the patient and/or wait list for a fatigue management clinic, whereas others were recruited via University networks of staff and students. The stated inclusion criteria were: >18 yrs, had any chronic condition, and were ambulatory – however it is not clear how it was determined if participants experienced fatigue (especially for the participants recruited via University networks) other than asking if they had received fatigue management advice or not. Please clarify how potential participants were assessed for the presence of fatigue as part of determining eligibility for the study. If the presence of fatigue was not assessed as part of the eligibility criteria for participation this would be a potential limitation – especially since the levels of fatigue were not similar between the two groups.

4. Please provide more information about the tailored fatigue advice that was provided ie. Was it based on a particular approach to fatigue management? What sorts of advice was provided/topics covered?

5. It states on page 6, line 140 that 'we will first conduct a cross-sectional study comparing individuals who have received fatigue management advice (in a fatigue clinic in the UK)...' However it is not clear if participants who were recruited through the 'university networks' who had previously received fatigue advice had received their advice from the same fatigue clinic? If not, was the fatigue advice similar for those who attended the fatigue clinic and those who were recruited through university networks? E.g. did the 'university network' participants also receive tailored advice about fatigue from a multi-disciplinary team of health professionals?

5. The method for calculating activity fluctuations has been clearly described, however it is not clear if the method used is one that has been previously validated/published. Please provide a reference/s for the calculation used if possible.

**Reviewer**	**2**
**Name**	**Hayes, Lawrence D**
**Affiliation**	**Lancaster University Medical School**
**Date**	**19-Sep-2025**
**COI**	**None**

Thank you for giving me the opportunity to review the manuscript "Unravelling the Diversity of Pacing Behaviours in Adults with Chronic Conditions: A Cross-Sectional Study"

I have tried to make the comments as constructive as possible, to enhance the manuscript where possible. Please feel free to come back with questions should anything be unclear.

Dr Lawrence Hayes, Lancaster University Medical School

general comments:

-is 7 days sufficient to determine 'activity fluctuations'? If PEM occurs 2 times per month for example, one week will be drastically different to the next week.

-'visualize' (US Spelling), but 'behaviour' (UK spelling). check the whole manuscript for consistency.

keywords:

consider adding 'energy management' which is the NICE wording for ME/CFS pacing.

strengths/limitations:

-a key limitation is that there is very limited evidence (i.e. no RCTs) that shows pacing is effective, so advising on something with limited evidence may be erroneous.

abstract:

-"Individuals with chronic conditions often experience fatigue and need to pace their activities to manage this fatigue while maintaining an active lifestyle" - is there evidence they maintain an active lifestyle? I would consider ending this sentence after the word fatigue.

-consider naming the questionnaires if word count allows (i.e. IPAQ, EuroQoL).

-"No difference in activity fluctuations during the day was found" - by activity fluctuations, is this some measure of variance? This is very important to explain to the reader, how this was arrived at, as the variable is emphasised often in the abstract.

-If wording allows, describe how PA was categorised into these patterns (1-5).

introduction:

-"sustained PA is crucial for managing chronic conditions, improving overall health and alleviating fatigue [8,9]." If wording allows it could also be useful for the reader that you include rehabilitation studies such as these: https://pubmed.ncbi.nlm.nih.gov/35236727/ ; https://pubmed.ncbi.nlm.nih.gov/37586104/ which shown exercise rehab improves chronic condition symptomology (despite how this may be received by patient groups).

-I think it could be worth including in the introduction that activity pacing evidence may be different by condition (i.e. MS more than ME), and include a small section (even one sentence) outlining that not all studies report benefits of activity pacing (e.g. The PACE Trial [highly controversial I know, but still the only RCT in ME/CFS]). Our group has published a meta-analysis in ME/CFS for example: https://www.tandfonline.com/doi/full/10.1080/21641846.2024.2433390#d1e633. Very few studies, very mixed results.

-I think it would also be useful to the reader to explain that activity pacing has minimal concrete steps for administration, is sometimes objective (with technology), and sometimes objective (spoons).

-"...recognising that fatigue is common across different conditions [1,6]." It could help the reader to give some examples of these conditions.

-"While some studies have explored activity pacing interventions [10,14,25,26]…" For a large RCT, our preprint (https://www.researchsquare.com/article/rs-5951040/v1) examined efficacy of technology-enabled pacing in long covid. I think this is one of the largest studies of pacing, so it may be worth including this (and possibly with a nod to technology generally [including the Visible app {I know this is not a suitable research app, but has a large consumer base]}).

-would it be possible somewhere in the introduction to explain what you mean by 'activity fluctuations'?

-"Our hypothesis is..." change to 'was' - past tense.

"fatigue management advice" - is this pacing advice? If not, fine. But if it is, try to use the same terminology throughout.

methods:

-"individuals who have received..." change to had perceived (do this for have nots as well).

-Please check the sample size calculation. If using rmANOVA and the interaction for inferences, the sample size may be 18, but isn't that per group?

-Is it appropriate for participants to self-report height and mass?

-Please correct me if I have misunderstood, but I am interpreting the activity fluctuation as the SD of cpm (of the vector) for each hour. This hourly SD value is then expressed per time period (6 hour epoch). My question is how do the 6 values (each hour), become 1 value? (6 hour epoch)? are they summed, averaged, or something else?

-The statistical analysis section (t-tests, regressions, Fishers, mixed model) does not match the sample size calc (rmANOVA). Please correct. https://bmcmedresmethodol.biomedcentral.com/articles/10.1186/s12874-022-01819-y may be helpful

results:

- As height and mass was self-reported, I suggest running sensitivity analysis without BMI (or height/weight) included, given known reporting biases.

--"a significant positive association between device-based PA and average activity fluctuation during the day for the whole sample (β=47.31; p=<0.001; 95%CI [37.08-57.54])." p cannot be equal to AND less than. remove =. Also, for the ease of reading, when 'device based PA' is given, does that mean total PA? i.e. more active people have more activity fluctuations?

- figure 1 needs y axes labels

-please ensure units are reported in all instances (even if arbitrary). For example, device-based PA is often written but is this METS?

-I would argue that two decimal places for all units is unnecessary. E.g. HRQoL and others are measured to the whole number.

-"demonstrates the highest score in perceived risk of overactivity (8.5(2.50))" inconsistent decimal places.

-table 1 has inconsistent decimal places between groups for mass, age, valid days.

-table 1 - report % to the whole number.

-table 3 has different decimal places to other tables. as suggested, i would default to the whole number for ease.

-for education, is there a way to internationalise this? i.e. 'sixth form college' i presume is quite specific wording to the UK. 'Undergraduate university' would imply they have not graduated. bachelors?

discussion:

This section seems excessively long. Could it be shortened?

VERSION 1 - AUTHOR RESPONSE

Reviewer 1

Comment 3: The manuscript is well written. The background information is clear, relevant and justifies the aim of the study. The aims are clearly stated. The methods are mostly thorough, with established outcome measures utilized relevant to the study aims. There are several areas related to participant recruitment and eligibility that need further explanation/clarification (see comments below). The results are clearly documented with good use of tables and figures. The discussion is in-depth and appropriately draws on relevant literature. The study strengths and limitations are appropriately described (with potentially another couple of limitations to include depending on responses to my comments/queries below). The conclusions are appropriate and does not overstate the study findings. **Response of the authors:** We thank you for your positive comments and structured feedback.

Comment 4: Please provide more information about how the potential participants from the fatigue clinic were invited by clinicians. Were all eligible participants invited? Or were only some selected to invite? If so, how were these people selected? How many people were invited in total? If there is potential for selection bias this needs to be included in the study limitations. **Response of the authors:** We thank the reviewer for this comment and clarification. All individuals attending the fatigue clinics or on the waiting list who met the inclusion criteria were eligible to be invited. Clinicians distributed information sheet letters to all eligible patients; no additional selection criteria were applied beyond the study's inclusion and exclusion criteria. We do not have exact figures for the total number of individuals approached, as the recruitment was conducted through a combination of methods (information letters distributed in fatigue clinics and a university-wide email) where precise tracking of the total reach was

not feasible. An estimated 80-120 information letters were distributed to potential participants through the fatigue clinics, while a recruitment email with the study information sheet was sent to all students and staff members at the university at the time. We have now added this information to the manuscript. The following has been added to the manuscript "Specifically, clinicians distributed an information letter to all eligible potential participants attending the fatigue clinics or on the waiting list; no additional selection criteria were applied beyond the study's inclusion and exclusion criteria. An estimated 80-120 information sheet letters were distributed to potential participants through the fatigue clinics. " (Page 9, Lines 193-196).

Comment 5: Was there any data collected regarding how long ago the participants had received the fatigue advice? If it was a long time ago this could affect implementation of the advice if participants forget key information over time. **Response of the authors:** Thank you for this important point. The participants were currently receiving fatigue management advice at the time of the study. We collected data on the duration for which participants had been receiving this advice, and this variable was accounted for in our analysis. We therefore believe that the concern regarding potential recall bias due to elapsed time since receiving the advice does not apply in this context.

Comment 6: The study sample included adults with chronic conditions who experience fatigue. Some participants were recruited from the patient and/or wait list for a fatigue management clinic, whereas others were recruited via University networks of staff and students. The stated inclusion criteria were: >18 yrs, had any chronic condition, and were ambulatory – however it is not clear how it was determined if participants experienced fatigue (especially for the participants recruited via University networks) other than asking if they had received fatigue management advice or not. Please clarify how potential participants were assessed for the presence of fatigue as part of determining eligibility for the study. If the presence of fatigue was not assessed as part of the eligibility criteria for participation this would be a potential limitation – especially since the levels of fatigue were not similar between the two groups. **Response of the authors:** Thank you for this important observation. We would like to clarify that experiencing fatigue was indeed part of the inclusion criteria for the study (added now - Page 9, Lines 207-208). All potential participants were required to self-report experiencing fatigue in addition to having been diagnosed with a chronic condition. This applied to participants recruited through both the fatigue management clinics and the University.

We acknowledge that the two groups differed significantly in their Fatigue Severity Scale (FSS) scores (6.47 vs. 4.44). However, it is worth noting that even participants in the no fatigue management advice group had a mean FSS score of 4.44, which exceeds the established clinical cut-off point of 4.0 on the FSS. This cut-off is widely used in the literature to indicate the presence of clinically significant fatigue. Therefore, while the groups differed in fatigue severity, participants in both groups were, on average, experiencing clinically meaningful levels of fatigue, supporting the appropriateness of the sample for the purposes of this study.

Comment 7: Please provide more information about the tailored fatigue advice that was provided ie. Was it based on a particular approach to fatigue management? What sorts of advice was provided/topics covered? **Response of the authors:** We appreciate this comment. As described in the manuscript, the tailored fatigue management advice provided at the clinic was tailored and was delivered by several healthcare professionals, including health psychologists, physiotherapists, occupational therapists, and a medical consultant. Activity pacing was one of the strategies the healthcare professionals were focusing on. This has been added in the manuscript "Page 8, Line 188". Unfortunately, as the fatigue management advice was delivered by the clinics independently of the research team, we do not have

access to further detailed information regarding the specific content or protocol of each individual session. It was real world input, not following a protocol, but personalised according to patients' needs and tailored to them.

Comment 8: It states on page 6, line 140 that 'we will first conduct a cross-sectional study comparing individuals who have received fatigue management advice (in a fatigue clinic in the UK)...' However it is not clear if participants who were recruited through the 'university networks' who had previously received fatigue advice had received their advice from the same fatigue clinic? If not, was the fatigue advice similar for those who attended the fatigue clinic and those who were recruited through university networks? E.g. did the 'university network' participants also receive tailored advice about fatigue from a multi-disciplinary team of health professionals? **Response of the authors:** Thank you for raising this point. We would like to clarify that participants recruited through University networks who reported having received fatigue management advice were not necessarily attending the same fatigue clinic as those recruited directly from the clinic's patient or waiting list. As such, we cannot confirm whether the fatigue advice received by these participants was identical in content or delivery to that provided at the specific fatigue clinic involved in the study. However, it is important to note that the primary aim of our study was to compare individuals who had received any form of fatigue management advice with those who had not, rather than to evaluate the effectiveness of a specific fatigue management programme. The variability in the source and nature of the advice received may, in fact, enhance the generalisability of our findings, as it reflects the diverse range of fatigue management support that individuals with chronic conditions may access in real-world settings. This has been added in the methods "Participants recruited through University networks who reported having received fatigue management advice may have received this advice from different clinics or healthcare providers, and the content of the advice received was not standardised across participants." Page 9, Lines 201-204

Comment 9: The method for calculating activity fluctuations has been clearly described, however it is not clear if the method used is one that has been previously validated/published. Please provide a reference/s for the calculation used if possible. **Response of the authors:** We appreciate this comment. We acknowledge that the specific method used for calculating activity fluctuations has not been validated. The ActiGraph wGT3X-BT device used in this study is a validated instrument for the assessment of physical activity. Furthermore, as noted in the manuscript, activity fluctuations or activity variability has been referenced in the literature as an accelerometer-derived measure of an aspect of activity pacing, and similar methods of calculation have been used in older previous studies, which we have cited accordingly. We recognise that the lack of a validated method for quantifying activity variability is a limitation and have acknowledged this in the limitations section "while the ActiGraph wGT3X-BT is a validated device for assessing physical activity, the method used to calculate activity fluctuations has not been formally validated, and further research is needed to establish standardised approaches for quantifying activity variability using accelerometer-derived data." Page 29, Lines 640-642

Reviewer 2

General Comments:
Comment 10: is 7 days sufficient to determine 'activity fluctuations'? If PEM occurs 2 times per month for example, one week will be drastically different to the next week. **Response of the authors:** Thank you for this thoughtful comment. A 7-day monitoring period is widely used and accepted in

accelerometry research, as it captures a full weekly cycle including both weekdays and weekends, and is generally considered sufficient to obtain a reliable estimate of habitual physical activity patterns. This duration is also commonly recommended in the literature to balance data reliability with participant burden, which is a particularly important consideration in populations experiencing fatigue due to chronic conditions, where prolonged monitoring periods may negatively impact compliance.
However, we acknowledge that a 7-day period may not capture less frequent fluctuations in activity, such as those associated with post-exertional malaise episodes that may occur on a less regular basis. Further research from our research group could investigate the use of longer monitoring periods alongside the validation of accelerometer-derived measures for quantifying activity fluctuations (comment 9). We have included this as a limitation in the limitations section. "while a 7-day accelerometry monitoring period is widely accepted for capturing habitual physical activity patterns, it may not sufficiently capture activity fluctuations, such as those associated with post-exertional malaise episodes, and future research should explore longer monitoring durations." Page 29, Lines 643-645

Comment 11: 'visualize' (US Spelling), but 'behaviour' (UK spelling). check the whole manuscript for consistency. **Response of the authors:** Thank you for pointing out this inconsistency. We have thoroughly reviewed the manuscript and ensured that British English spelling is used consistently throughout.

keywords:
Comment 12: consider adding 'energy management' which is the NICE wording for ME/CFS pacing. **Response of the authors:** We have added it now.

strengths/limitations:
Comment 13: a key limitation is that there is very limited evidence (i.e. no RCTs) that shows pacing is effective, so advising on something with limited evidence may be erroneous. **Response of the authors:** Thank you for this important comment. We agree that the evidence from randomised controlled trials on the effectiveness of activity pacing is currently limited. However, we would like to clarify that the aim of our study is not to advise on activity pacing, but rather to explore it as a construct in individuals with chronic conditions who experience fatigue. Furthermore, there is a growing body of research that has investigated activity pacing both quantitatively and qualitatively, including studies exploring the perspectives of both patients and healthcare professionals, which collectively suggest the potential value of pacing. Our study contributes to this developing evidence base, rather than making recommendations regarding pacing interventions.

Abstract:

-Comment 14: "Individuals with chronic conditions often experience fatigue and need to pace their activities to manage this fatigue while maintaining an active lifestyle" - is there evidence they maintain an active lifestyle? I would consider ending this sentence after the word fatigue. **Response of the authors:** We have ended the sentence after the word fatigue, as suggested.

Comment 15: consider naming the questionnaires if word count allows (i.e. IPAQ, EuroQoL). Thank you for this suggestion. We have included the names of the questionnaires in the abstract. "Secondary outcomes included fatigue (FSS), engagement in activity pacing, perceived risk of overactivity, quality

of life (FACT-G), self-regulation of PA (PASR-12), and self-reported PA (IPAQ)." Pages 2-3, Lines 42-44

Comment 16: "No difference in activity fluctuations during the day was found" - by activity fluctuations, is this some measure of variance? This is very important to explain to the reader, how this was arrived at, as the variable is emphasised often in the abstract. **Response of the authors:** Thank you for this important point. We have revised the abstract to clarify that activity fluctuations is the standard deviation of accelerometer-derived vector magnitude counts per minute. Page 2, Line 41

Comment 17: If wording allows, describe how PA was categorised into these patterns (1-5). **Response of the authors:** Thank you for this comment. We have added a brief description in the abstract to clarify that the five pacing patterns were identified through visual inspection of individual activity profiles across the week. Page 3, Lines 48-49

Introduction:

Comment 18: "sustained PA is crucial for managing chronic conditions, improving overall health and alleviating fatigue [8,9]." If wording allows it could also be useful for the reader that you include rehabilitation studies such as these: https://pubmed.ncbi.nlm.nih.gov/35236727/ ; https://pubmed.ncbi.nlm.nih.gov/37586104/ which shown exercise rehab improves chronic condition symptomology (despite how this may be received by patient groups). **Response of the authors:** Thank you for this suggestion and for providing these relevant references. We agree that it would be valuable to highlight for the reader that rehabilitation and exercise-based interventions have demonstrated benefits for chronic condition symptomology. We have added the following sentences: "Rehabilitation studies have demonstrated that targeted exercise interventions could improve symptomology in chronic conditions, including improvements in breathlessness, quality of life and functional capacity following inspiratory muscle training in post-COVID-19 recovery and exercise-based rehabilitation programmes." Page 4, Lines 81-84

Comment 19: I think it could be worth including in the introduction that activity pacing evidence may be different by condition (i.e. MS more than ME), and include a small section (even one sentence) outlining that not all studies report benefits of activity pacing (e.g. The PACE Trial [highly controversial I know, but still the only RCT in ME/CFS]). Our group has published a meta-analysis in ME/CFS for example: https://www.tandfonline.com/doi/full/10.1080/21641846.2024.2433390#d1e633. Very few studies, very mixed results. **Response of the authors:** Thank you for this valuable suggestion and for sharing your group's recent meta-analysis. We agree that it is important to acknowledge in the introduction that the evidence for activity pacing varies across conditions and that not all studies report consistent benefits. We have added a sentence in the introduction referencing the mixed evidence. "However, the evidence for activity pacing is not consistent across all conditions. A recent meta-analysis of pacing in ME/CFS found that while pacing exerted a large beneficial effect on fatigue, effects on physical function and pain were trivial." Page 5, Lines 104-106

Comment 20: I think it would also be useful to the reader to explain that activity pacing has minimal concrete steps for administration, is sometimes objective (with technology), and sometimes objective (spoons). **Response of the authors:** Thank you for this suggestion. We agree that it would be useful to clarify this for the reader. Given that activity pacing is a multidimensional concept that is tailored to each individual's needs, baseline, environment, and goals, it does not lend itself to a prescriptive set of concrete steps for administration. Furthermore, pacing can be assessed in different ways. For example,

device-based, through technology such as accelerometers, and subjectively, through self-management approaches such as Spoon Theory, where individuals 'budget' their perceived energy across daily tasks. This variability in both the delivery and assessment of activity pacing further underscores the need for research that explores how individuals implement pacing in their daily lives. We have added the following sentences in the introduction to acknowledge this. "Due to this multidimensional nature and the need to tailor pacing to each individual's specific needs and goals, there are currently no standardised concrete steps for its administration. Moreover, pacing can be assessed in different ways, ranging from device-based measures using accelerometry to subjective self-management approaches such as Spoon Theory, where individuals budget their perceived energy across daily tasks". Page 5, Lines 95-100

Comment 21: "...recognising that fatigue is common across different conditions [1,6]." It could help the reader to give some examples of these conditions. **Response of the authors:** Thank you for the suggestion. We have added the following: "...including fibromyalgia and multiple sclerosis." Page 6, Line 119

Comment 22: "While some studies have explored activity pacing interventions [10,14,25,26]…" For a large RCT, our preprint (https://www.researchsquare.com/article/rs-5951040/v1) examined efficacy of technology-enabled pacing in long covid. I think this is one of the largest studies of pacing, so it may be worth including this (and possibly with a nod to technology generally [including the Visible app {I know this is not a suitable research app, but has a large consumer base]}). **Response of the authors:**

Comment 23: -would it be possible somewhere in the introduction to explain what you mean by 'activity fluctuations'? **Response of the authors:** We have now explained in the introduction as follows "Activity fluctuations refer to the variability in physical activity intensity, reflecting how an individual distributes their activity across different time periods." Page 5, Lines 111-112

Comment 24: "Our hypothesis is..." change to 'was' - past tense. **Response of the authors:** We changed that. Thank you.

Comment 25: "fatigue management advice" - is this pacing advice? If not, fine. But if it is, try to use the same terminology throughout. **Response of the authors:** Thank you for this comment. Activity pacing is a fatigue management strategy. Since individuals received tailored advice that may have encompassed a range of strategies beyond activity pacing alone, the term "fatigue management advice" better reflects the broader nature of the advice received.

Methods:

Comment 26: "individuals who have received..." change to had perceived (do this for have nots as well). **Response of the authors:** We have corrected that.

Comment 27: -Please check the sample size calculation. If using rmANOVA and the interaction for inferences, the sample size may be 18, but isn't that per group? **Response of the authors:** We

Comment 28: -Is it appropriate for participants to self-report height and mass? **Response of the authors:** Thank you for raising this point. Self-reported height and body mass were used in this study due to practical constraints, as data collection was conducted remotely. Self-reported height and weight have been shown to be reasonably accurate and are widely used in epidemiological and health research, particularly in studies where in-person assessments are not feasible.

Comment 29: -Please correct me if I have misunderstood, but I am interpreting the activity fluctuation as the SD of cpm (of the vector) for each hour. This hourly SD value is then expressed per time period (6 hour epoch). My question is how do the 6 values (each hour), become 1 value? (6 hour epoch)? are they summed, averaged, or something else? **Response of the authors:** Thank you for this question and for allowing us to clarify. The activity fluctuation was calculated as follows: first, the standard deviation (SD) of the vector magnitude counts per minute (cpm) was computed for each individual hour within each time period (morning: 06:00-12:00; afternoon: 12:00-18:00; evening: 18:00-00:00), producing 6 hourly SD values per time period per day. These 6 hourly SD values were then averaged to produce a single value for each time period per day. Finally, these daily values were averaged across the 7 monitored days to produce a weekly average for each time period. We have revised the methods section to make this calculation procedure more explicit and hope this addresses the reviewer's concern. "to calculate the activity fluctuations, the standard deviation (SD) of counts per minute (cpm) was first computed for each individual hour using the vector magnitude, a variable that combined the 3 axes in one outcome defined as $\sqrt{(x^2 + y^2 + z^2)}$. This was calculated across three time periods during the day (morning: 06:00 AM–12:00 PM; afternoon: 12:00 PM–06:00 PM; evening: 06:00 PM–12:00 AM), producing 6 hourly SD values per time period per day. These 6 hourly SD values were then averaged to produce a single activity fluctuation value for each time period per day. Finally, these daily values were averaged across the 7 monitored days (Monday–Sunday) to produce a weekly average for each time period. The measure of activity fluctuations during an average day in the week provides insight into the distribution of activities over the day, which contributes to characterising an important element of activity pacing behaviour." Page 13, Lines 287-297

Comment 30: -The statistical analysis section (t-tests, regressions, Fishers, mixed model) does not match the sample size calc (rmANOVA). Please correct. https://bmcmedresmethodol.biomedcentral.com/articles/10.1186/s12874-022-01819-y may be helpful. **Response of the authors:** We thank the reviewer for this observation. We acknowledge that the power calculation reported in the original manuscript was incorrectly specified. The correct sample size estimate based on that original calculation was 20 per group (40 total). However, upon reflection, we recognise that reporting a formal power calculation is not appropriate for this study. As stated in the title, this is an exploratory, cross-sectional investigation and, to our knowledge, the first to collect device-based accelerometer data on activity pacing behaviours in adults with chronic conditions. Given the absence of prior comparable studies from which to derive meaningful effect size estimates, and the exploratory nature of the research objectives, a formal a priori sample size calculation was not difficult to conduct. We have therefore removed the power calculation from the revised manuscript and replaced it with a justification with the study's exploratory design. Page 10, Lines 210-212

Results:

Comment 31: As height and mass was self-reported, I suggest running sensitivity analysis without BMI (or height/weight) included, given known reporting biases. **Response of the authors:** We thank the reviewer for this valuable suggestion. In response, we conducted sensitivity analyses in which self-reported BMI was excluded as a covariate from all models, including the interaction analyses. The results of these sensitivity analyses were highly consistent with the primary analyses: the direction, magnitude, and statistical significance of the associations remained unchanged. This indicates that our findings are robust and not driven by potential bias related to self-reported height and weight. The results of these sensitivity analyses are presented in Supplementary Table 2. A sentence is added in the methods mentioning that we performed sensitivity analyses without self-reported BMI, and a sentence

in the results is added mentioning the results of these analyses. Page 16, Lines 366-367; Page 20, Lines 441-443

Comment 32: "a significant positive association between device-based PA and average activity fluctuation during the day for the whole sample (β=47.31; p=<0.001; 95%CI [37.08-57.54])." p cannot be equal to AND less than. remove =. Also, for the ease of reading, when 'device based PA' is given, does that mean total PA? i.e. more active people have more activity fluctuations? **Response of the authors:** We appreciate your comment. We have now removed the '='.
The "device-based PA" does not refer to total physical activity. As described in the methods section, device-based physical activity was operationalised as the percentage of time spent in moderate-to-vigorous physical activity (MVPA), derived from the ActiGraph wGT3X-BT accelerometer data using the Freedson cut-point algorithm (≥1952 counts per minute). Therefore, higher values of device-based PA reflect a greater proportion of time spent in moderate-to-vigorous intensity activities specifically, rather than overall activity volume.

Comment 33: figure 1 needs y axes labels. **Response of the authors:** Thank you for this comment. We would like to clarify that a y-axis label has been included in Figure 1; however, rather than repeating the label on each individual subfigure, a single shared y-axis label ("Standard deviation of Vector Magnitude (counts/min)") has been placed on the left-hand side of the figure to apply to all subfigures collectively. fThis approach was used to maximise the readability of each individual panel, as including a separate y-axis label on each of the 29 subfigures would have significantly reduced the available plotting area.

Comment 34: please ensure units are reported in all instances (even if arbitrary). For example, device-based PA is often written but is this METS? **Response of the authors:** We have corrected that.

Comment 35: I would argue that two decimal places for all units is unnecessary. E.g. HRQoL and others are measured to the whole number. **Response of the authors:** We have corrected that

Comment 36: "demonstrates the highest score in perceived risk of overactivity (8.5(2.50))" inconsistent decimal places. **Response of the authors:** We have corrected that.

Comment 37: table 1 has inconsistent decimal places between groups for mass, age, valid days.
Response of the authors: We have corrected that.

Comment 38: -table 1 - report % to the whole number.
Response of the authors: We have corrected that.

Comment 39: table 3 has different decimal places to other tables. as suggested, i would default to the whole number for ease.
Response of the authors: We have corrected that.

Comment 40: for education, is there a way to internationalise this? i.e. 'sixth form college' i presume is quite specific wording to the UK. 'Undergraduate university' would imply they have not graduated. Bachelors?
Response of the authors: Thank you for this suggestion. We have revised the educational categories to be more internationally recognisable. Specifically, "sixth form college" has been replaced with "post-

secondary", "undergraduate university" has been replaced with "Bachelor's degree", and "postgraduate" has been replaced with "postgraduate degree". See in Table 1.

discussion:
Comment 41: This section seems excessively long. Could it be shortened?
Response of the authors: We have shortened the discussion as suggested.

VERSION 2 - REVIEW

**Reviewer**	**2**
**Name**	**Hayes, Lawrence D**
**Affiliation**	**Lancaster University Medical School**
**Date**	**30-Apr-2026**
**COI**	

Thank you for taking the time to revise the manuscript. The paper is (is my opinion) clearer to the reader. However, two comments have been left without a response.

Comment 22: "While some studies have explored activity pacing interventions [10,14,25,26]…" For a large RCT, our preprint (https://www.researchsquare.com/article/rs-5951040/v1) examined efficacy of technology-enabled pacing in long covid. I think this is one of the largest studies of pacing, so it may be worth including this (and possibly with a nod to technology generally [including the Visible app {I know this is not a suitable research app, but has a large consumer base]}). Response of the authors: BLANK

This paper has now been published in Nature Comms in 2026, so reflects contemporary literature: https://pmc.ncbi.nlm.nih.gov/articles/PMC12864992/

Comment 27: -Please check the sample size calculation. If using rmANOVA and the interaction for

inferences, the sample size may be 18, but isn't that per group? Response of the authors: We BLANK

Once these have been addressed, I see no reason why the article cannot be accepted.

VERSION 2 - AUTHOR RESPONSE

Comment 22 - "While some studies have explored activity pacing interventions [10,14,25,26]…" For a large RCT, our preprint (https://www.researchsquare.com/article/rs-5951040/v1) examined efficacy of technology-enabled pacing in long covid. I think this is one of the largest studies of pacing, so it may be worth including this (and possibly with a nod to technology generally [including the Visible app {I know this is not a suitable research app, but has a large consumer base]}). Response of the authors: BLANK

This paper has now been published in Nature Comms in 2026, so reflects contemporary literature: https://pmc.ncbi.nlm.nih.gov/articles/PMC12864992/
Response of the authors: We thank the reviewer for this suggestion and for sharing this work. Indeed, referencing such a large RCT of technology-enabled pacing strengthens our introduction. We have revised the relevant paragraph to include a reference to Sanal-Hayes et al. (2026). We have also included a brief acknowledgement of the growing role of technology in pacing delivery, including consumer-facing platforms. The paragraph has been updated accordingly. (Page 7, Lines 150-155).

Comment 27 - Please check the sample size calculation. If using rmANOVA and the interaction for inferences, the sample size may be 18, but isn't that per group? Response of the authors: We BLANK
Response of the authors: We thank the reviewer for this comment. The reviewer is correct, the sample size of 17 reported in the previous submitted manuscript referred to the number per group, not the total sample. The correct estimate based on the original calculation was 20 per group (40 total), accounting for anticipated attrition. However, as detailed in our response to Comment 30 (in the previous response document), we have removed the power calculation from the revised manuscript, as a formal a priori sample size calculation is not appropriate for this exploratory study. We have replaced it with a justification consistent with the study's exploratory design. (Page 10, Lines 213-215).
